# Identification of TBX2 and TBX3 variants in patients with conotruncal heart defects by target sequencing

**DOI:** 10.1186/s40246-018-0176-0

**Published:** 2018-09-17

**Authors:** Huilin Xie, Erge Zhang, Nanchao Hong, Qihua Fu, Fen Li, Sun Chen, Yu Yu, Kun Sun

**Affiliations:** 1 Department of Pediatric Cardiovascular, Xin Hua Hospital, School of Medicine, Shanghai Jiao Tong University, Shanghai, 200092 China; 2 Medical Laboratory, Shanghai Children’s Medical Center, School of Medicine, Shanghai Jiao Tong University, Shanghai, 200127 China; 3 Department of Pediatric Cardiology, Shanghai Children’s Medical Center, School of Medicine, Shanghai Jiao Tong University, Shanghai, 200127 China

**Keywords:** TBX2, TBX3, Variant, Conotruncal heart defects, Target sequencing

## Abstract

**Background:**

Conotruncal heart defects (CTDs) are heterogeneous congenital heart malformations that result from outflow tract dysplasia; however, the genetic determinants underlying CTDs remain unclear. Increasing evidence demonstrates that dysfunctional TBX2 and TBX3 result in outflow tract malformations, implying that both of them are involved in CTD pathogenesis. We screened for TBX2 and TBX3 variants in a large cohort of CTD patients (*n* = 588) and population-matched healthy controls (*n* = 300) by target sequencing and genetically analyzed the expression and function of these variants.

**Results:**

The probably damaging variants p.R608W, p.T249I, and p.R616Q of TBX2 and p.A192T, p.M65L, and p.A562V of TBX3 were identified in CTD patients, but none in controls. All altered amino acids were highly conserved evolutionarily. Moreover, our data suggested that mRNA and protein expressions of TBX2 and TBX3 variants were altered compared with those of the wild-type. We screened PEA3 and MEF2C as novel downstream genes of TBX2 and TBX3, respectively. Functional analysis revealed that TBX2R608W and TBX2R616Q variant proteins further activated HAS2 promoter but failed to activate PEA3 promoter and that TBX3A192T and TBX3A562V variant proteins showed a reduced transcriptional activity over MEF2C promoter.

**Conclusions:**

Our results indicate that the R608W and R616Q variants of TBX2 as well as the A192T and A562V variants of TBX3 contribute to CTD etiology; this was the first association of variants of TBX2 and TBX3 to CTDs based on a large population.

**Electronic supplementary material:**

The online version of this article (10.1186/s40246-018-0176-0) contains supplementary material, which is available to authorized users.

## Background

Conotruncal heart defects (CTDs) are a group of complex congenital heart malformations with an estimated prevalence of 0.1% of live births and roughly 10–25% in congenital heart defects (CHD) [1] and are caused by abnormal development of the outflow tract (OFT) in embryo or abnormal configuration and arrangement of the ventricle, septal tissue, and large vessels; CTDs include the tetralogy of Fallot (TOF), persistent truncus arteriosus (PTA), double outlet of right ventricle (DORV), transposition of the great arteries (TGA), single atrium (SA), single ventricle (SV), and others. CTDs commonly occur in infants and children and are chief causes of infant death and childhood disability. Most CTD patients require catheter-based or surgical interventions early in life because without intervention, the diseases could lead to poor quality of life with mental and physical retardation, severe cardiac arrhythmias, heart failure, and even sudden death. Although therapeutic regimens have increased survival into adulthood in patients with CTDs, the morbidity and mortality rates remain high in survivors [2, 3]. In addition, CTDs induce heavy economic burdens on society, especially as the survival rates and the number of adults living with CTDs increase [4].

The development of the OFT in the embryonic stage is an elaborate regulatory process, which includes the formation and development of the secondary heart field (SHF) and cardiac neural crest (CNC), during which any abnormal factor of inheritance or environment can lead to abnormal proliferation, differentiation, or migration of SHF and CNC cells, thereby causing CTDs [5–7]. Increasing studies demonstrated that genetic factors played primary roles in pathogenesis of CTDs, but the genetic determinants underlying CTDs remain unclear [8, 9].

In genetics, many transcription factors are recognized as major contributors to normal cardiac morphogenesis, including the T-box family of transcription factors [10]. TBX2 and TBX3 are members of the T-box family of transcription factors that are important for early cardiogenic lineage development as well as formation of chambers and the conduction system [11]. At embryonic day 8–10 mouse heart, Tbx2 was detected in the non-chamber myocardium, which included the atrioventricular canal (AVC), inner curvature, inflow tract, and OFT [12]. Tbx2 expression patterns during chick heart development are consistent with that of mouse [13]. Morphological defects of the heart were observed in Tbx2 knock-out mouse embryos, including abnormal atrioventricular morphology and outflow tract septation defects [14]. Mutations in TBX3 specifically cause ulnar-mammary syndrome [15]. TBX3 is most closely related to TBX2. Tbx3-null mouse embryos have atrioventricular alignment and OFT defects and various kinds of cardiac malformation, such as DORV and TGA. In addition, Tbx3 is involved in multiple signaling pathways that regulate OFT morphogenesis and SHF proliferation [16]. Both Tbx2 and Tbx3 express embryo mesoderm adjacent to CNC and the SHF [16–18]. Accordingly, overexpression/low expression or abnormal function of TBX2 and TBX3 causes heart defects and thus play an important role in heart development.

Current studies for TBX2 and TBX3 have typically focused on gene knock-out animal models to observe phenotype and explore mechanism; however, they have not investigated whether genetic variants were involved in pathogenesis in populations of CTD patients. Only Pang et al. reported variants (g.59477201C>T, g.59477347G>A, g.59477353delG, and g.59477371G>A) located at the TBX2 gene promoter in a cohort of 324 patients with ventricular septal defects, and the variants reduced the transcriptional activities of the TBX2 gene promoter [19]. Therefore, identifying rare variants of TBX2 and TBX3 in large CTD cohort is required urgently.

Here, we show several rare heterozygous variants of TBX2 and TBX3 by target sequencing in a cohort of 588 CTD patients without 22q11.2 deletion, but none in population-matched healthy controls. Our data shows that these variants alter mRNA and protein expression of TBX2 and TBX3. We screen PEA3 and MEF2C as novel downstream target genes of TBX2 and TBX3, respectively. Function analyses reveal that the variants of TBX2 or TBX3 may regulate PEA3, MEF2C, and HAS2 (the known downstream gene of TBX2) promoting CTD incidence, first defining the connection between TBX2/TBX3 variants and CTDs and further elucidating the genetic pathogenesis of CTDs.

## Results

### The variants of TBX2 and TBX3 identified in CTD patients

**Fig. 1:**
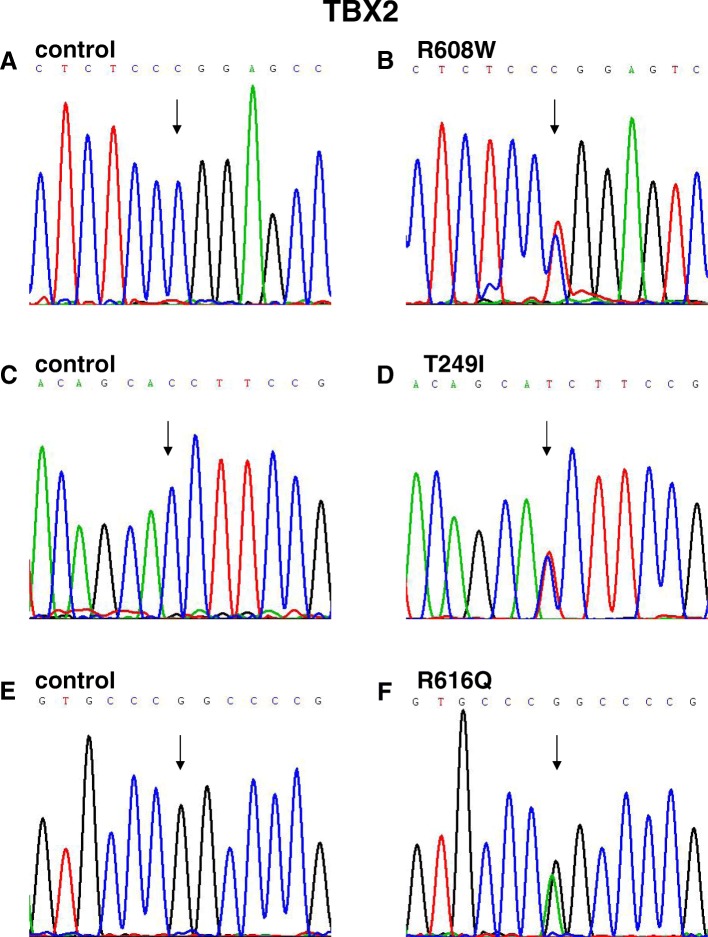
Sequence chromatograms of TBX2 missense variants in patients and controls. **a**, **c**, and **e** Chromatograms of normal controls. **b**, **d**, and **f** Chromatograms of the three heterozygous variants. Arrows show heterozygous nucleotide changes

**Fig. 2:**
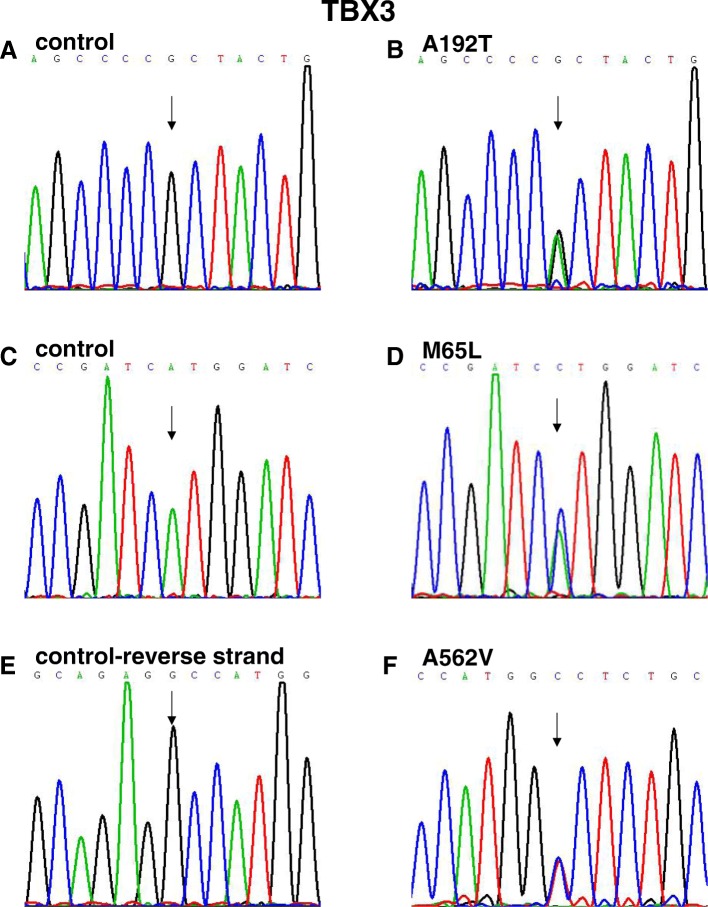
Sequence chromatograms of TBX3 missense variants in patients and controls. **a**, **c**, and **e** Chromatograms of normal controls. **b**, **d**, and **f** Chromatograms of the three heterozygous variants. Arrows show heterozygous nucleotide changes

We found variants of TBX2 and TBX3 through target sequencing in 588 CTD patients and identified three variants of TBX2 in seven patients and three variants of TBX3 in six patients, including TOF, TGA, SA, and SV (Table 1). The variants of TBX2 were p.R608W, p.T249I, and p.R616Q (Fig. 1b, d, and f), and the variants of TBX3 were p.A192T, p.M65L, and p.A562V (Fig. 2b, d, and f). The p.R608W, p.T249I, and p.R616Q variants of TBX2 and p.A192T and p.M65L variants of TBX3 in both control and case groups were in HWE; the p.A562V variant of TBX3 in control group was in HWE, but in case group, it was not in HWE (Additional file 1: Table S1). However, these variants all led to amino acid substitutions and were predicted to be damaging as per SIFT, Polyphen-2, or Mutation Taster (Table 1).

**Table 1:** Clinical information and variant characteristics of TBX2 and TBX3 in patients with CTDs

Patient	Gender	Age	Cardiac phenotype	Gene	Location in gene	Function	Nucleotide change	Amino acid change	dbSNP ID	SIFT	Mutation taster	PolyPhen-2	ExAC allele frequency
1	F	4 months	TOF	TBX2	exon7	Missense	1822C>T	R608W	rs764896880	0.01	Disease causing	1.000	1.726e−05
2	F	8 months	TOF	TBX2	exon3	Missense	746C>T	T249I	rs778075071	0.02	Disease causing	0.967	6.633e−05
3	F	5 months	SV	TBX2	exon3	Missense	746C>T	T249I	rs778075071	0.02	Disease causing	0.967	6.633e−05
4	F	5 years	SV	TBX2	exon3	Missense	746C>T	T249I	rs778075071	0.02	Disease causing	0.967	6.633e−05
5	M	6 months	TOF	TBX2	exon7	Missense	1847G>A	R616Q	rs191930922	0.01	Disease causing	0.865	0.0008162
6	F	3 years	SA	TBX2	exon7	Missense	1847G>A	R616Q	rs191930922	0.01	Disease causing	0.865	0.0008162
7	M	1 year	TGA	TBX3	exon6	Missense	574G>A	A192T	rs768160499	0.11	Disease causing	1.000	2.471e−05
8	M	1 year	TOF	TBX3	exon1	Missense	193A>C	M65L	/	0.56	Disease causing	0.734	8.675e−06
9	M	5 months	TOF	TBX3	exon7	Missense	1685C>T	A562V	rs201325654	0.1	Disease causing	0.849	0.002761
10	M	6 months	TOF	TBX3	exon7	Missense	1685C>T	A562V	rs201325654	0.1	Disease causing	0.849	0.002761
11	F	5 months	TOF	TBX3	exon7	Missense	1685C>T	A562V	rs201325654	0.1	Disease causing	0.849	0.002761
12	F	6 months	TOF	TBX3	exon7	Missense	1685C>T	A562V	rs201325654	0.1	Disease causing	0.849	0.002761
13	M	4 years	TGA	TBX3	exon7	Missense	1685C>T	A562V	rs201325654	0.1	Disease causing	0.849	0.002761

*F* female, *M* male, *CTDs* conotruncal heart defects, *TOF* tetralogy of Fallot, *SA* single atrium, *SV* single ventricle, *TGA* transposition of the great arteries

### Alignment of multiple TBX2 and TBX3 protein sequences and display of the structure of human TBX2 and TBX3 protein

**Fig. 3:**
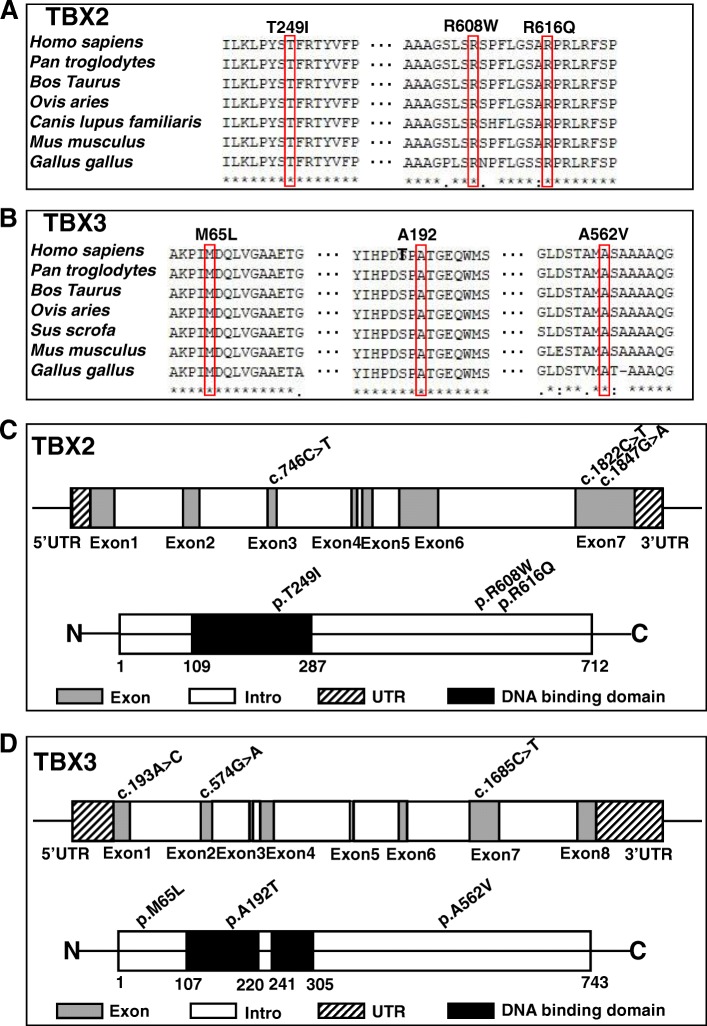
Conservation and distribution of TBX2 and TBX3 variants. **a**, **b** Alignments of TBX2 and TBX3 protein among different species. All variants were highly conserved in vertebrates. **c**, **d** Diagram of the TBX2 and TBX3 gene exons and protein with location of variants identified in this study

All variation sites in this study were highly conserved in vertebrates, as shown in multiple TBX2 and TBX3 protein alignments (Fig. 3a, b), indicating that these variants were very important and might result in TBX2 and TBX3 gene function alterations. The human TBX2 spans 3396 bp, and has been mapped to chromosome 17q23, which is composed of seven exons and six introns (20). The T-box DNA-binding domain of TBX2 is located at amino acids 109–287 (Fig. 3c). The human TBX3 mapped to chromosome 12q24, spans 4814 bp and is composed of eight exons and seven introns. The T-box DNA-binding domain of TBX3 is located at amino acids 107–220 and 241–305 (Fig. 3d) (Uniprot: http://www.uniprot.org/).

### Detection of TBX2 and TBX3 variant expression

**Fig. 4:**
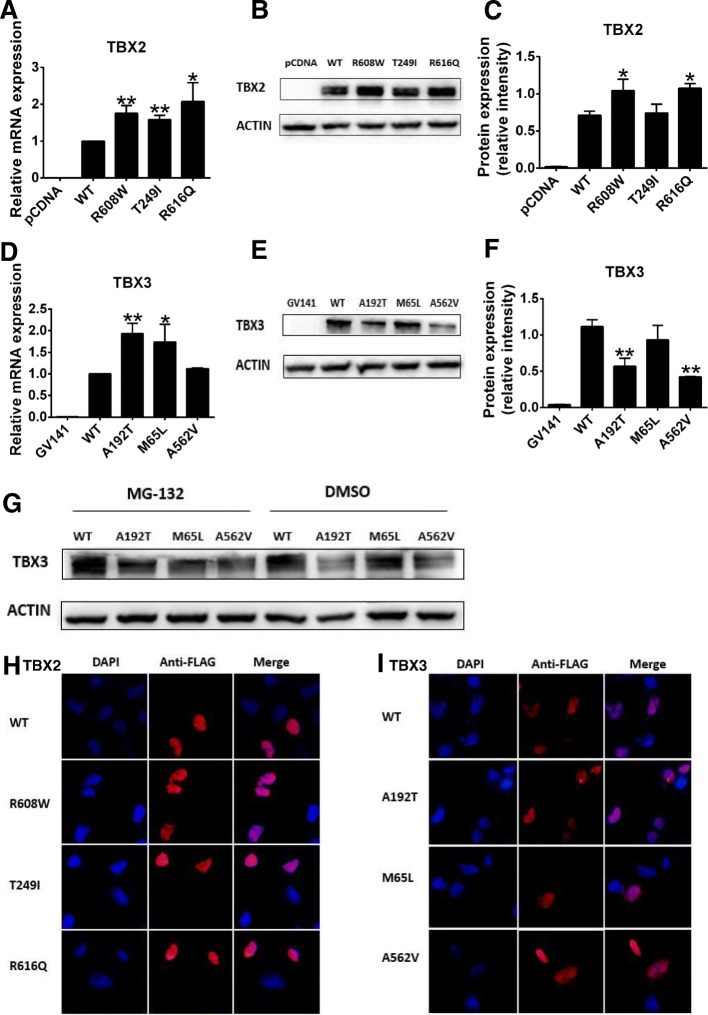
mRNA abundance, protein expression level, and subcellular localization of TBX2 and TBX3. Plasmids were transfected into HEK 293T cells and harvested. **a**, **d** Relative mRNA expression of the blank vector, wild-type plasmid, and variants of TBX2 and TBX3 (*n* = 3). GAPDH was used as an internal control. **b**, **e** Western blot analysis of the blank vector, wild-type plasmid, and variants of TBX2 and TBX3. Actin was used as an internal control. **c**, **f** Density quantitation of TBX2 and TBX3 variant protein expression as shown in **b** and **e** (*n* = 3). **g** Western blot analysis of TBX3 variant protein degradation through the ubiquitin-proteasome pathway. MG-132 was the proteinase inhibitor and DMSO was used as control. **h**, **i** Representative images of immunofluorescence staining of TBX2 and TBX3 variants and wild-type proteins. *P < 0.05, **P < 0.01 versus WT; data represented here are obtained from three biological replicates

To investigate whether the expression of the TBX2 and TBX3 variants were altered, we performed quantitative RT-PCR and Western blot. Quantitative RT-PCR analysis revealed that mRNA expression of R608W, T249I, and R616Q variants of TBX2 (Fig. 4a) and A192T and M65L variants of TBX3 (Fig. 4d) were greater than that of the group of the wild-type plasmid (*P* < 0.05). On Western blot, protein expression of R608W and R616Q variants (Fig. 4b, c) was distinctly greater than that of the wild-type TBX2 (*P* < 0.05), consistent with the mRNA expression of these two TBX2 variants; in contrast, protein expression of A192T and A562V variants (Fig. 4e, f) were notably lower than that of the wild-type TBX3 (*P* < 0.05), indicating that TBX3 variants might lead to protein degradation. Tbx3 is associated with SUMOylation (SUMO, small ubiquitin-related modifier) that may be a conserved mechanism controlling Tbx3 activity [21, 22]. Therefore, we observed the effect of ubiquitin-proteasome degradation and found that the reduction of A192T variant protein expression was rescued after adding the protease inhibitor, suggesting that the A192T variant decreased TBX3 protein stability by ubiquitin-proteasome degradation (Fig. 4g).

### Nuclear localization of TBX2 and TBX3 variants

To detect the cellular distribution of TBX2 and TBX3 variant proteins, we carried out immunofluorescence assays that demonstrated that all the TBX2 and TBX3 variant proteins were expressed in the nucleus, as were wild-type TBX2 and TBX3 proteins (Fig. 4h, i). The result suggested that these variant proteins might affect TBX2 or TBX3 gene function through other mechanisms.

### Screening of downstream target genes of TBX2 and TBX3

**Fig. 5:**
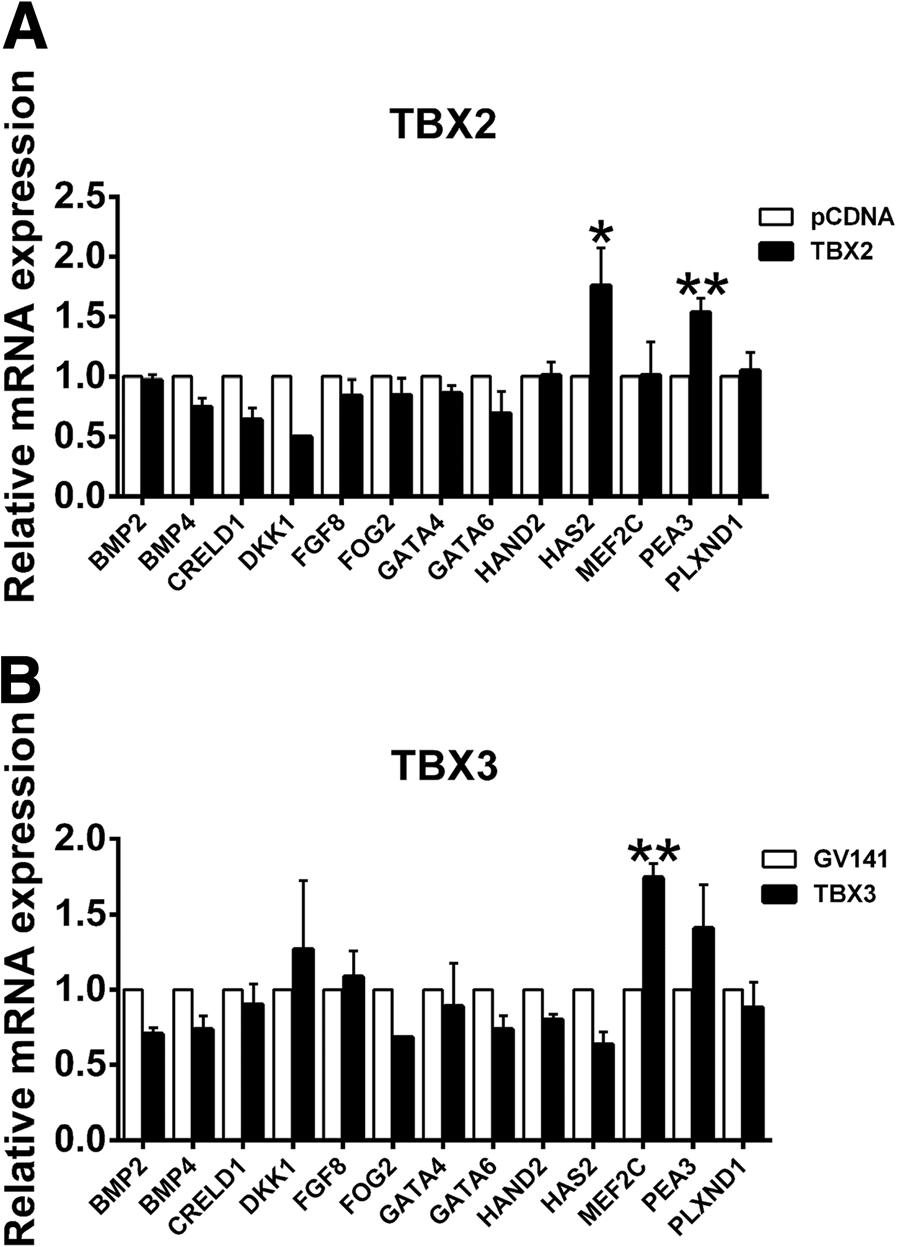
Screening of downstream target genes related to CTDs of TBX2 or TBX3. **a**, **b** Relative mRNA expression of candidate downstream target genes affected by wild-type TBX2 or TBX3 transfection (*n* = 3). GAPDH was used as an internal control. *P < 0.05, **P < 0.01 versus pCDNA or GV141; data represented here are obtained from three biological replicates

We selected several genes related to CTDs or OFT development from previous studies (BMP2 [13], BMP4 [13], FGF8 [23, 24], HAS2 [25], PEA3 [16]), and the MalaCards database (CRELD1, DKK1, FOG2, GATA4, GATA6, HAND2, MEF2C, PLXND1) and then screened the possible downstream genes of TBX2/TBX3 by observing mRNA expression alterations of these target genes after TBX2/TBX3 overexpression (Fig. 5a, b). The results showed that TBX2 activated HAS2 and PEA3, while TBX3 activated MEF2C (*P* < 0.05), indicating that these were downstream genes of TBX2/TBX3 in CTD incidence.

### Transcriptional activity of TBX2 and TBX3 variant proteins

**Fig. 6:**
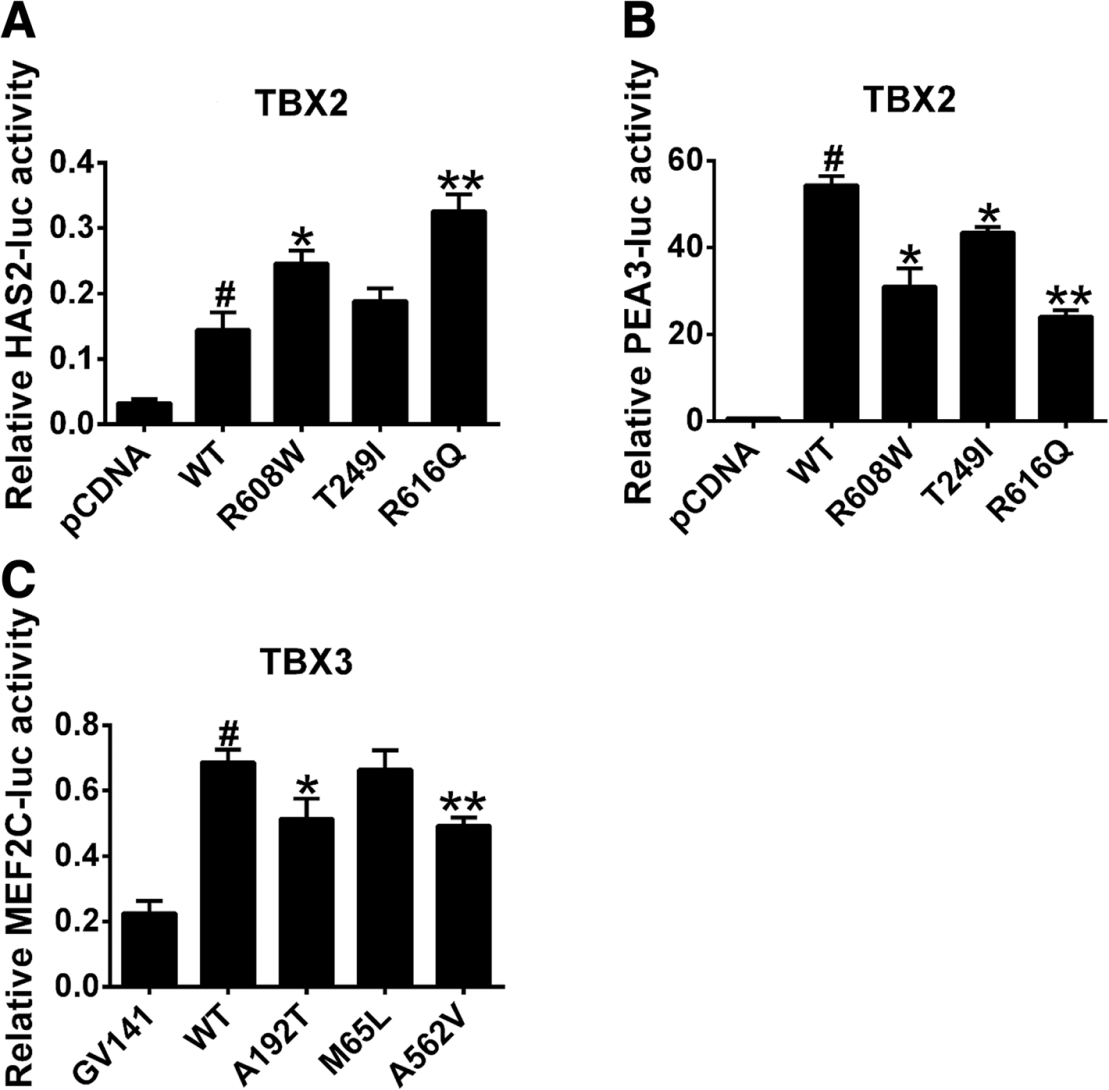
The activity of HAS2, PEA3, and MEF2C luciferase reporter genes regulated by TBX2 or TBX3. HEK 293T cells that were co-transfected with vector or wild-type or variant plasmid and a luciferase reporter; transcriptional activity was measured by a luciferase reporter gene detection system. pRL-TK was used as an internal control. **a**, **b** Luciferase activity of HAS2, PEA3 promoter regulated by blank vector, and wild-type and variants of TBX2 (*n* = 3). **c** Luciferase activity of MEF2C promoter regulated by blank vector, wild-type, and variants of TBX3 (*n* = 3). ^#^*P* < 0.01 versus pCDNA or GV141 and *P < 0.05, **P < 0.01 versus WT; data represented here are obtained from three biological replicates

To evaluate the ability of the TBX2/TBX3 variants to regulate downstream genes, we constructed luciferase reporter genes for human HAS2, MEF2C, and PEA3 promoters selected from the mRNA expression screening and co-transfected them with wild-type or variants of TBX2/TBX3. Compared with wild-type TBX2, R608W and R616Q variant proteins were able to activate HAS2 promoter up to approximately 1.7 times (*P* < 0.05) and 2.6 times (*P* < 0.01), respectively (Fig. 6a), whereas the transcriptional activity of R608W and R616Q variant proteins to activate PEA3 promoter reduced by 45% (*P* < 0.05) and 55% (*P* < 0.01), respectively (Fig. 6b). Compared with wild-type TBX3, A192T, and A562V variant proteins showed a reduced transcriptional activity over MEF2C promoter (Fig. 6c). Therefore, R608W and R616Q variants of TBX2 and A192T and A562V variants of TBX3 may affect TBX2/TBX3 regulation on downstream target genes through transcriptional activity alteration, thereby promoting CTD incidence.

### Expression of TBX3 protein in the human embryo

**Fig. 7:**
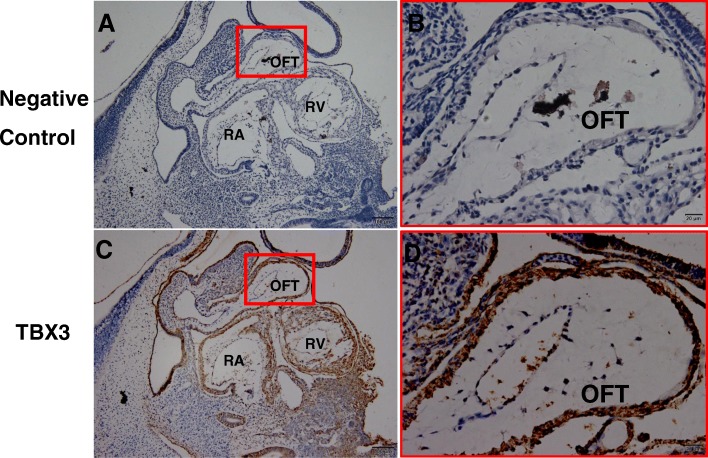
Immunohistochemistry of TBX3 in human embryos in Carnegie 13 stage. **a**, **b** Negative control. **c**, **d** Wild-type TBX3. Scale bar 20 μm

TBX2 has been reported as being expressed at the AVC and OFT of the heart in animals and human embryos [12]. However, TBX3 expression has not been identified in the human embryo; therefore, we selected human embryos in Carnegie 13 stage, which is the crucial period of the OFT formation to carry out immunohistochemistry. The results showed that TBX3 was expressed in the nucleus in the OFT (Fig. 7c, d), indicating that TBX3 might have a function in the development of the OFT.

**Fig. 8:**
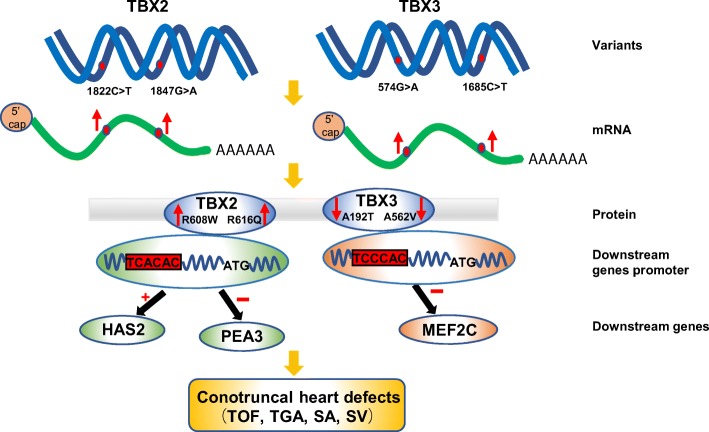
The diagram of the regulation TBX2 and TBX3 variants involved in CTD pathogenesis

We show the illustration summarizing our results and claims (Fig. 8).

## Discussion

Identifying rare variants of TBX2 and TBX3 in a large CTD cohort is required urgently; our study found six rare heterozygous variants of TBX2 and TBX3 in 13 patients from 588 sporadic patients with CTDs using target sequencing that included p.R608W, p.T249I, and p.R616Q variants of TBX2 and p.A192T, p.M65L, and p.A562V variants of TBX3, all of which were all highly conserved based on multiple sequence alignment, suggesting that these variants might have important biological functions. Among these variants, TBX3M65L was the novel variant first reported, and TBX2R608W had not been reported formerly in East Asians. The other four variants were reported in Ensemble (http://ensemblgenomes.org/); their functions had never been studied before. All these variants were predicted as damaging as per SIFT, Mutation Taster or Polyphen-2. Moreover, TBX2R608W, TBX2R616Q, TBX3A192T, and TBX3A562V showed altered expression and function compared with wild-type TBX2/TBX3. Nevertheless, none of the six variants affected the subcellular expression of TBX2 and TBX3 in nucleus, and it is possible that these variants were not located in the critical region that influenced the nuclear distribution of TBX2 and TBX3 [26].

Tbx2 and Tbx3 have common downstream target genes that have been identified as chamber myocardium-specific genes, including connexin 40 (Cx40), connexin 43 (Cx43), and natriuretic precursor peptide type A (Nppa) [27, 28]. Locally repressing these chamber specific genes is required for the formation of non-chamber myocardium and induction of the development of the AVC, inflow tract, and OFT [29, 30]. However, mRNA expression of Cx40, Cx43, and Nppa in HEK293 cells showed no difference after TBX2 or TBX3 overexpression (data not shown) probably due to the limitations of the cell line or the experimental model.

We screened 12 genes selected from previous studies and the MalaCards database (http://www.malacards.org/) as candidates for TBX2 or TBX3 downstream genes and found that mRNA expression of HAS2 and PEA3 in HEK293 cells were elevated substantially after TBX2 overexpression, while TBX3 transfection upregulated MEF2C abundance significantly. The activity of HAS2, MEF2C, and PEA3 luciferase reporter genes was dramatically increased by wild-type TBX2/TBX3 compared with that of blank vectors, providing further evidence that the expression of HAS2, PEA3, and MEF2C was regulated by TBX2 or TBX3. HAS2 has conserved T-box binding sites among promoter regions and encodes hyaluronan synthases 2, the major enzyme responsible for hyaluronan (HA) production in the heart [31], and it is expressed in the OFT [20]. However, excess HA deposition may cause hemodynamic alterations and may block cardiomyocyte differentiation. Tbx2 contributes to the expansion of the extracellular matrix (ECM) and epithelial-mesenchymal transformation (EMT) by inducing Has2 myocardial expression and increasing HA deposition to drive endocardial cushion formation and altered cardiogenic lineage specification in embryonic hearts [25]. Overexpression of HAS2 leading to HA deposition may hinder cardiomyocyte differentiation. We found that TBX2 variants activated HAS2 and caused CTDs and were similar to this report. PEA3 (polyomavirus enhancer activator 3, currently called ETV4, ETS variant 4) is a transcription factor belonging to the PEA3 family and is involved in chromosomal translocation associated with Ewing tumors, whose overexpression promotes cell proliferation, motility, and invasion. The evidences suggest that PEA3 plays a role in cellular proliferation, differentiation, and migration [32, 33]. A previous study reported that Pea3 was associated with Tbx3 involved in regulating OFT morphogenesis [16]. However, in the present study, TBX3 did not affect mRNA expression of PEA3, whereas TBX2 markedly increased PEA3 mRNA abundance and promoter activity; meanwhile, the wild-type TBX2 protein was able to significantly activate its promoter and the TBX2 variant proteins failed to activate its promoter, indicating that PEA3 was a novel downstream gene of TBX2 causing CTDs. TBX2 contributes to oncogenesis and cell cycle regulation [34], analogous to the functions of PEA3 [32, 33]. Therefore, we inferred that TBX2 and PEA3 may engage in crosstalk during cell cycle regulation and that more studies are needed to determine whether they take part in cardiac development through the regulation that these pathways require. MEF2C (MEF2 polypeptide C) is a member of the MADS box transcription enhancer factor 2 (MEF2) family of proteins, which play roles in myogenesis. Previous studies demonstrated that MEF2C is required for proper OFT alignment [35]. Moreover, MEF2C has also been involved in congenital OFT defects in humans [36]. Our results showed that MEF2C was a novel downstream gene of TBX3 and that variants of TBX3 might regulate MEF2C to cause CTDs.

Previous studies reported Tbx2 and Tbx3 were transcriptional repressors [22, 23]. In contrast, TBX2 and TBX3 in our study were activators; we showed that wild-type TBX2 activated the HAS2 gene promoter and wild-type TBX3 activated the MEF2C gene promoter. On the one hand, TBX2 has the capacity to activate a promoter including multiple T-box elements by a weak activation domain located within the T-box [37]. We presumed that the regulating domain of TBX3 resembles that of TBX2. On the other hand, it can also be explained by the presumption that transcriptional repression of TBX2 and TBX3 may depend on the cell line and the primary cardiomyocyte; therefore, our results are limited by the single cell line we used.

Dysfunction of Tbx2 and Tbx3 leading to heart defects has been verified in animals, providing strong evidence that TBX2 and TBX3 are significant for human cardiac morphogenesis and the underlying etiology of CTDs. There were some limitations to our study; for example, the lack of parental samples limited our ability to study the genetic background of these variants. Nevertheless, this study provided the first genetic evidence of an association between malfunctioning TBX2 and TBX3 and CTDs based on a large population, contributing to prenatal diagnosis and prenatal consultation in favor of the early prophylaxis and allele-specific therapy of CTDs.

## Conclusions

We found that the variants of TBX2 and TBX3 contributed to the occurrence of CTDs, and we explored the interesting downstream genes of TBX2 and TBX3 to illuminate the mechanisms of CTD etiology. Meanwhile, our findings open new fields of investigation into CTD genetic pathogenesis.

## Methods

### Study population

Our study population included 588 sporadic nonsyndromic CTD patients diagnosed by echocardiogram, cardiac catheterization, or surgery from Shanghai Xin Hua Hospital. The participants are from the Chinese Han population and included 388 males and 200 females, with ages ranging from 0.1 to 17 years (Table 2). Patients with known syndromes or chromosomal abnormalities, such as 22q11.2 deletion, were excluded from our study. The controls were 300 healthy children without heart disease. Both patient and control groups gave informed consent for inclusion, and then, peripheral blood was collected for DNA extraction. The genomic DNA of participants was extracted by using the QIAamp DNA Blood Mini Kit (QIAGEN, Germany) following the manufacturer’s instructions and was then stored at − 80 °C.

**Table 2:** Cardiac diagnoses for study population of patients with CTDs

Diagnoses	Number	Gender/number	Age
TGA	90	F18M72	1 day–16 years
TOF	234	F83M151	1 month–13 years
DORV	98	F32M66	1 month–17 years
PA/VSD	97	F36M61	3 months–12 years
IAA	13	F7M6	7 days–1 year
PTA	10	F5M5	3 days–2 years
SA/SV	46	F19M27	1 month–13 years
Total	588	F200M388	1 day–17 years

*TGA* transposition of the great arteries, *TOF* tetralogy of Fallot, *DORV* double outlet of right ventricle, *PA/VSD* pulmonary atresia with ventricular septal defect, *IAA* interruption of aortic arch, *PTA* persistent truncus arteriosus, *SA* single atrium, *SV* single ventricle, *F* female, *M* male

### Target sequencing and variant analysis

Target sequencing was performed using the Illumina HiSeq 2000 platform for variants in TBX2 (GenBank accession number NC_000017.11, NM_005994.3) and TBX3 (GenBank accession number NC_000012.12, NM_016569.3). The candidate variants were validated by Sanger sequencing, and the primers were designed for PCR amplification of TBX2 and TBX3. To predict the effects of nonsynonymous variants, we used several bioinformatics criteria including SIFT (http://sift.jcvi.org/www/SIFT_enst_submit.html), Mutation Taster (http://www.mutationtaster.org/), and Polyphen-2 (http://genetics.bwh.havard.edu/pph2/). Amino acid substitutions were predicted as damaging when the score was ≤ 0.05 in SIFT or ≥ 0.85 in Polyphen-2. In our study, variants with a minor allele frequency (MAF) < 0.5% were defined as rare [38].

### Multiple TBX2 and TBX3 protein sequence alignment

TBX2 and TBX3 protein sequences from *Homo sapiens* (human), *Mus musculus* (house mouse), *Gallus gallus* (chicken), *Bos taurus* (cattle), *Canis lupus familiaris* (dog), *Pan troglodytes* (chimpanzee), and *Sus scrofa* (pig) were downloaded from NCBI (https://www.ncbi.nlm.nih.gov/protein/) and were aligned with ClustalX software to confirm the conservation of TBX2 and TBX3 sequences.

### Plasmid construction and site-directed mutagenesis

The TBX2 and TBX3 cDNA plasmid was purchased from Genomeditech. Mutated primers were designed to amplify human TBX2 and TBX3 cDNA according to the protocol provided by the QuikChange SiteDirected Mutagenesis Kit (Stratagene, USA), and then, variant TBX2 cDNAs were cloned into a pCDNA3.1-3xFlag vectors while variant TBX3 cDNAs were cloned into GV141-3xFlag vectors. For recombining luciferase reporter plasmid, a 5′-flanking region of downstream gene promoter was subcloned into Kpn I and Bgl II sites of the pGL3 luciferase reporter-basic vector (Promega, USA).

### Cell cultures and transfection

HEK 293T cells (Human embryonic kidney cells) were maintained in Dulbecco’s modified Eagle’s medium (HyClone, USA) with 10% fetal bovine serum (MP Biomedicals, USA) and 1% penicillin-streptomycin (Gibco, USA). pcDNA3.1-3xFlag-TBX2 and GV141-3xFlag-TBX3 including wild-type and variants were transfected into 293T cells with FuGene HD (Promega, USA) according to the manufacturer’s protocol after seeding 24 h.

### Quantitative RT-PCR

Plasmids were transfected into HEK 293T cells that were seeded in 12-well plates. Cells were harvested 36 h after transfection. Total RNA was extracted with TRIzol reagent (Invitrogen, USA), and then, reverse transcription of cDNA was performed using Prime Script RT Master Mix (Takara, Japan) and was followed by quantitative RT-PCR using SYBR Premix Ex Taq (Takara, Japan) on an Applied Biosystems 7500 system (Applied Biosystems, USA). The relative quantification of expression was determined using the 2^-△△Ct method [39], and glyceraldehyde-3-phosphate dehydrogenase (GAPDH, human) was used as an internal control. Primer sequences of TBX2, TBX3, GAPDH, and candidate downstream genes are listed in Table 3.

**Table 3:** Sequences of the primers used for real-time quantitative PCR

Gene	Forward (5′→3′)	Reverse (5′→3′)
GAPDH	GGAGCGAGATCCCTCCAAAAT	GGCTGTTGTCATACTTCTCATGG
TBX2	CACGGCTTCACCATCCTAAAC	TGCGGAAGGTGCTGTAAGG
TBX3	GAGGCTAAAGAACTTTGGGATCA	CATTTCGGGGTCGGCCTTA
BMP2	GAGGTCCTGAGCGAGTTCGA	ACCTGAGTGCCTGCGATACA
BMP4	ATGATTCCTGGTAACCGAATGC	CCCCGTCTCAGGTATCAAACT
CRELD1	GCTCCTATGAGTGCCGAGAC	CTACACTTCTTACAGCGACCTG
DKK1	ATAGCACCTTGGATGGGTATTCC	CTGATGACCGGAGACAAACAG
FGF8	GACCCCTTCGCAAAGCTCAT	CCGTTGCTCTTGGCGATCA
FOG2	GGCCTGAAAATCTGAGCTGC	CAGTCGTCTGTCTCAACTCCA
GATA4	CGACACCCCAATCTCGATATG	GTTGCACAGATAGTGACCCGT
GATA6	CTCAGTTCCTACGCTTCGCAT	GTCGAGGTCAGTGAACAGCA
HAND2	CGCCGACACCAAACTCTCC	TCGCCATTCTGGTCGTCCT
HAS2	CTCATCATCCAAAGCCTGTT	GCTGGGTCAAGCATAGTGTC
MEF2C	CCAACTTCGAGATGCCAGTCT	GTCGATGTGTTACACCAGGAG
PLXND1	AATGGGCGGAACATCGTCAAG	CGAGACTGGTTGGAAACACAG
PEA3	GAGAAACCTCTGCGACCATT	GCCCGTCCAGGCAATGAAAT

### Western blot

HEK 293T cells were transfected with 1 μg of wild-type and variant plasmid DNA. Cells were harvested 48 h after transfection. For protein degradation experiments, HEK 293T cells were cultured in the presence of 10 mM MG132 solution dissolved in DMSO for 8–10 h before harvest. Then, cells were lysed in RIPA lysis buffer (Beyotime, China) with PMSF (1:100). The proteins were subjected to 10% SDS-PAGE and were then transferred onto nitrocellulose membranes (Millipore, USA) and immunostained with rabbit anti-FLAG antibody (1:1000, Sigma-Aldrich, USA) and mouse anti-β-actin antibody (1:5000, Sigma-Aldrich, USA) at 4 °C overnight. The membranes were incubated with horseradish peroxidase-conjugated anti-rabbit secondary antibody (1:10000) and anti-mouse secondary antibody (1:10000). Immobilon Western Chemiluminescent HRP Substrate (Millipore, USA) was used for chemiluminescent immunodetection.

### Luciferase assays

HEK 293T cells were transfected with 200 ng of wild-type or variant plasmid DNA, 200 ng of luciferase reporter plasmid, and 8 ng of an internal control reporter plasmid (pRL-TK) (Promega, USA) in a 48-well plate. The luciferase activity was measured on the Dual-Glo luciferase assay system (Promega, USA) following the manufacturer’s protocol after 48 h.

### Immunofluorescence assay

HEK 293T cells were seeded onto a 24-well plate covered with slips coated with poly-L-lysine (0.1 mg/mL) for 24 h and were then transfected with wild-type or variant plasmid DNA. Cells were harvested 24 h after transfection. Cells were incubated with rabbit anti-Flag antibody (1:100, Sigma-Aldrich, USA) diluted in PBS containing 5% BSA and 0.1% Triton X-100 at 4 °C overnight and followed by incubation with Cy3-conjugated goat anti-rabbit secondary antibody (1:250). Cell nuclei were stained by 4,6-diamidino-2-phenylindole (DAPI) (Vector Laboratories, USA). A Leica SP8 microscope was used for image analysis.

### Tissue collection and immunohistochemistry

Human embryos of Carnegie 13 stage were acquired after medical termination of pregnancy at Shanghai Xin Hua Hospital. The medical ethics committee of Xin Hua Hospital approved the study. Embryos were fixed overnight in 4% paraformaldehyde in PBS, embedded in paraffin, and sectioned at a thickness of 7 μm. For immunolocalization of TBX3, paraffin sections were incubated with a primary rabbit anti-TBX3 antibody (1:50, Protein-tech), followed by horseradish peroxidase-conjugated secondary anti-rabbit antibody and DAB (Abcam, UK).

### Statistical analysis

Each assay was performed for three independent biological replicates. The data are shown as the mean ± standard deviation (SD). Statistical differences were evaluated by one-way ANOVA and two-tailed unpaired *t* test. A *P* value < 0.05 was considered statistically significant.

## Additional file

Additional file 1: **Table S1.** Frequencies of alleles and genotypes of TBX2 and TBX3 variants in CTD patients and controls. (DOCX 19 kb)

## Abbreviations

AVC: Atrioventricular canal
CNC: Cardiac neural crest
CTDs: Conotruncal heart defects
HA: Hyaluronan
OFT: Outflow tract
SHF: Secondary heart field
TBX2/3: T-box 2/3 [*Homo sapiens* (human)]
Tbx2/3: T-box 2/3 [*Mus musculus* (house mouse)]
