# Supplementary material for: Identification of TBX2 and TBX3 variants in patients with conotruncal heart defects by target sequencing
Source: Hum Genomics. 2018 Sep 17;12:44. doi: 10.1186/s40246-018-0176-0 (PMC6142335; doi:10.1186/s40246-018-0176-0)
Supplement: Supplementary file 1 — Table S1. Frequencies of alleles and genotypes of TBX2 and TBX3 variants in CTD patients and controls. (DOCX 19 kb) [file 40246_2018_176_MOESM1_ESM.docx]

Additional file 1: Table S1. Frequencies of alleles and genotypes of TBX2 and TBX3 variants in CTD patients and controls.

| Gene | Cases n=588 No (%) | Control n=300 No (%) |
| --- | --- | --- |
| TBX2-1822C/T Genotype frequency | | |
| CC | 587 (99.8) | 300 (100) |
| CT | 1 (0.2) | 0 (0) |
| TT | 0 (0) | 0 (0) |
| TBX2-1822C/T Allele frequency | | |
| C | 1175 (99.9) | 600 (100) |
| T | 1 (0.1) | 0 (0) |
| P value | 1 | 1 |
| TBX2-746C/T Genotype frequency | | |
| CC | 585 (99.4) | 300 (100) |
| CT | 3 (0.6) | 0 (0) |
| TT | 0 (0) | 0 (0) |
| TBX2-746C/T Allele frequency | | |
| C | 1173 (99.7) | 600 (100) |
| T | 3 (0.3) | 0 (0) |
| P value | 1 | 1 |
| TBX2-1847G/A Genotype frequency | | |
| GG | 586 (99.6) | 300 (100) |
| GA | 2 (0.4) | 0 (0) |
| AA | 0 (0) | 0 (0) |
| TBX2-1847G/A Allele frequency | | |
| G | 1174 (99.8) | 600 (100) |
| A | 2 (0.2) | 0 (0) |
| P value | 1 | 1 |
| TBX3-574G/A Genotype frequency | | |
| GG | 587 (99.8) | 300 (100) |
| GA | 1 (0.2) | 0 (0) |
| AA | 0 (0) | 0 (0) |
| TBX3-574G/A Allele frequency | | |
| G | 1175 (99.9) | 600 (100) |
| A | 1 (0.1) | 0 (0) |
| P value | 1 | 1 |
| TBX3-193A/C Genotype frequency | | |
| AA | 587 (99.8) | 300 (100) |
| AC | 1 (0.2) | 0 (0) |
| CC | 0 (0) | 0 (0) |
| TBX3-193A/C Allele frequency | | |
| A | 1175 (99.9) | 600 (100) |
| C | 1 (0.1) | 0 (0) |
| P value | 1 | 1 |
| TBX3-1685C/T Genotype frequency | | |
| CC | 583 (99.1) | 300 (100) |
| CT | 4 (0.7) | 0 (0) |
| TT | 1 (0.2) | 0 (0) |
| TBX3-1685C/T Allele frequency | | |
| C | 1170 (99.5) | 600 (100) |
| T | 6 (0.5) | 0 (0) |
| P value | 0.013 | 1 |
